# The Standard Dictionary

**Published:** 1891-10

**Authors:** 


The Standard Dictionary of the English Language is a new and superior work which is just going through the press. It will embody many new principles in lexicography, and will contain nearly 2,200 pages; over 4,000 illustrations made especially for this work; 200,000 words; 70,000 more words than in any other single volume dictionary. Price, when issued, $12.00. At $7.00 to advance subscribers. Publishers, Funk & Wagnalls. New York, 18 and 20 Astor Place. London, 44 Fleet Street. Toronto, Canada, 86 Bay Street.

